# Uncovering Hidden Curricula: Use of Dark Humor in Anatomy Labs and its Implications for Basic Sciences Education

**DOI:** 10.1007/s40670-019-00912-0

**Published:** 2020-01-15

**Authors:** Angelique N. Dueñas, Karen Kirkness, Gabrielle M. Finn

**Affiliations:** Health Professions Education Unit, Hull York Medical School, John Hughlings Jackson Building, University Rd., Heslington, York, YO10 5DD UK

**Keywords:** Anatomy education, Humor, Dark humor, Hidden curriculum, Professionalism, Medical education

## Abstract

Humor is subjective within most settings, but within the anatomy laboratory, it is likely to be significantly more contentious. While humor may be considered a component of the hidden curriculum of medical education, it has yet to be studied specifically from a basic sciences perspective. This study sought to understand if, when, how, and why humor may be used in anatomy labs and the implications this may have in basic sciences education. A survey consisting of demographic and qualitative items was designed to sample widely from academics, students, and health professionals with anatomy laboratory experience. A total of 185 respondents, representing 9 countries participated following purposive sampling and snowball recruitment. Findings of significance were 72% of respondents who had experienced dark humor within the anatomy lab. Themes identified from free-text pertained to the use of internal and external barometers to ascertain the appropriateness of humorous remarks and the use of humor as a mechanism for diffusing stress. Polarity in responses concerning the acceptability of dark humor and rude mnemonics was also observed. This study highlighted that while dark humor may be a perceived tension release, many individuals make use of very specific internalized gauges to determine when and what humor may be appropriate. The data emphasized the need for not only future humanistic-focused anatomy but also basic sciences, education research, to better understand and have ideal educational experiences for all. Finally, this study provided further evidence of the impact of the hidden curriculum associated with the use of humor within educational and professional settings.

## Introduction

In recent years, there have been more humanistic considerations within the basic sciences, but particularly within anatomy education. Rightfully, the days when “cadaver antics” and “cadaver stories” were common enough to be considered rites of passage have largely dissipated from the modern anatomy lab [1, 2]. Furthermore, there has been a movement away from “detached concern” and viewing patients as objects by health professions, which has been mirrored in gross anatomy labs and within the education context [3]. It is not uncommon to hear of anatomical donors now referred to as “first teachers” or “first patients” [4, 5].

Many programs now incorporate ethics and humanities curriculum formally into anatomy education [6, 7]. For example, many medical schools include reflective writing tasks or donor-focused writing as a component of their formal anatomy requirements [4, 8, 9]. One pertinent example comes from a Finnish institution that has designed formal death and dying lectures for their medical students, run by an interdisciplinary team, including a psychiatrist, anatomist, and hospital pastor [10]. These lectures precede any gross lab experiences and have been generally well-received by students.

Alongside formalized curricula, there has also been an increase in the understanding of humanistic and emotional aspects associated with anatomy labs [11, 12]. Some research suggests that anatomy labs can be a “stressful” environment. Furthermore, for many students, the anatomy lab presents first experiences in extremely close proximity to death [3, 13, 14]. As Dinsmore et al. [14] note in their study, the students that report higher levels of associated stress make up a small percentage of the whole, and stress often dissipates with exposure. Some studies suggest that labs might prove more challenging compared with other academic demands, rather than being outright distressing [15–17].

Regardless of level or specific cause of stress, these studies do highlight that general anatomy lab associated stress is well documented. However, there is a paucity of research relating to how students, or staff, might cope with the stressors of gross anatomy lab experiences. One study of medical student experiences with dissection in the UK highlighted potential negative reactions as a small component of initial anatomy perceptions but does not explore how specifically such emotions were overcome [18]. Arráez-Aybar et al. [19] suggested that anxiety that is associated with anatomy lab experiences may be most strongly mediated and diminished simply with repeated exposure. But in a follow-up study investigating anatomy lab stress mediators, a large number of health professions students highlighted the potential of humor or jokes as a means to deal with lab associated anxiety [20]. Another study of medical students found anatomy labs to be a low stressor for students initially, but that a high percentage of students used humor to cope with stress and keep their dissection group on task [21]. Similar findings were presented by Kotzé and Mole [22] in a study that highlighted the benefits of peer talk, and also humor, to cope with aspects of death and dying, associated with dissection.

These anatomy-specific findings align with a subset of medical education research that suggested one of the most common coping mechanisms for acutely stressful and morbid medical experiences is dark humor [2, 23, 24]. Dark humor, often synonymous with black, gallows, or cynical humor, is described as a comic style that makes light of typically taboo subjects, normally considered painful to discuss [2]. Research shows that dark humor is a common element of medical practice, bringing together teams and helping individuals cope with traumatic and high-stress situations, often relating to illness, trauma, and death [25–28].

Yet, there have been no humor-focused studies in anatomy education, despite similar themes of death associated with gross anatomy. The findings that humor may be used as a coping mechanism in labs, as discussed above, have not been specifically addressed. Furthermore, there are no studies that the authors could find investigating the details of when and why humor is used specifically in anatomy labs, or the views of in-groups on the use of humor, particularly dark humor.

Thus, this study was designed with the aims of first understanding if and when humor is used in anatomy labs. Further, if humor is being used in labs, what are the general views on the perceived purpose of humor? Given the highly variable opinions on what constitutes “humor,” this study focused on black humor, synonymous with cynical, dark, or gallows humor. This type of humor was selected due to its definition of being humor that treats serious or possibly taboo subjects (such as death or working with human specimens) in a light or satirical fashion [2]. Given that previous studies in medical education have found dark humor to be a common coping mechanism, particularly in the face of trauma, death, and dying, the authors hypothesized that dark humor would be acknowledged as used in anatomy labs and justified as an “appropriate” means of coping with working with cadaveric materials.

## Methods

A 15-item, non-validated survey was designed to collect demographic and humor views from individuals with anatomy lab experiences. The majority of questions are related to if, when, and how much participants experienced the use of humor in anatomy lab settings. There were also follow-up items to ascertain why and when humor might be used in anatomy labs and an opportunity to respond to a hypothetical situation. Google’s survey software was implemented to disseminate the survey. This study was granted ethical approval by the Hull York Medical School Ethics Committee (Ref #18 34).

Data collection commenced in 3 phases. The survey was first piloted within the Hull York Medical School. After piloting, the survey was disseminated more widely, with a focus on UK and international recruitment. Participants were recruited via social media posts and virtual snowball sampling via email [29]. The only inclusion criteria for the survey was anatomy lab experience of any kind. Consent was implied through the completion of the survey.

Descriptive analysis for items was performed in Microsoft Excel®. All open-ended responses were anonymized from the rest of the data and provided to co-authors to code. Coding was conducted independently by two authors, before negotiating and agreeing on both final themes and illustrative quotes. Coding was conducted manually, using an inductive approach to thematically analyze free-text responses [30, 31]. Authors were reflexive in their qualitative approach [32, 33] by acknowledging their preconceived notions towards the research and personal research paradigms; all authors noted their experiences hearing dark humor and various other modes of humor in anatomy labs. However, the addition of frequency statements and a closed-ended question were used in an attempt to directly sample participants on their beliefs or experiences on humor in labs.

## Results

### Demographics and Experiences

A total of 185 participants completed the survey. Table 1 provides a complete summary of demographic data. Nine countries were represented, although the majority of the participants indicated the United Kingdom as their country of residence. A total of 62% of participants identified as female and the ages of participants ranged from 18 to 65 years old, although younger age groups were more greatly represented. There was also variability in the positions that respondents identified as, although the majority indicated that they were students in healthcare or anatomy programs.

**Table 1:** Participant demographic information

Demographics	Survey, *n* (%)
What country do you reside in?	
UK	120 (65%)
USA	32 (17%)
Canada	15 (8%)
Australia	8 (4%)
India	2 (1%)
Germany	1 (< 1%)
Hong Kong	1 (< 1%)
Jamaica	1 (< 1%)
New Zealand	1 (< 1%)
Did not disclose	4 (2%)
Which gender do you identify as?	
Female	114 (62%)
Male	71 (38%)
Prefer not to say/other	0 (0%)
What is your age? (years)	
18–22	59 (32%)
23–27	44 (24%)
28–32	24 (13%)
33–37	18 (10%)
38–42	16 (8%)
43–47	4 (2%)
48+	14 (8%)
Did not disclose	6 (3%)
Which of the following best describes your position?	
Anatomy academic staff/faculty	58 (31%)
Year 1–2 health care program student	32 (17%)
Year 3–5 health care program student	30 (16%)
Undergraduate student	18 (10%)
Master’s level student	16 (9%)
PhD level student	14 (8%)
Anatomy lab technical staff	5 (3%)
Health care program graduate	6 (3%)
Anatomy program graduate	4 (2%)
Anatomy research staff	2 (1%)

When asked to describe their general anatomy lab experience, 25% of participants reported a combination of prosection, dissection, and teaching. This was followed by 22% with prosection only experience. A total of 38% of respondents reported some combination of dissection experiences, either associated with prosection, teaching, forensic work, or other laboratory activities, such as histology. Five percent of participants reported teaching anatomy in some capacity with no further details, and 10% of participants did not disclose the details of their anatomy lab experiences.

### Frequency of Negative Lab and Humor Experiences

**Fig. 1:**
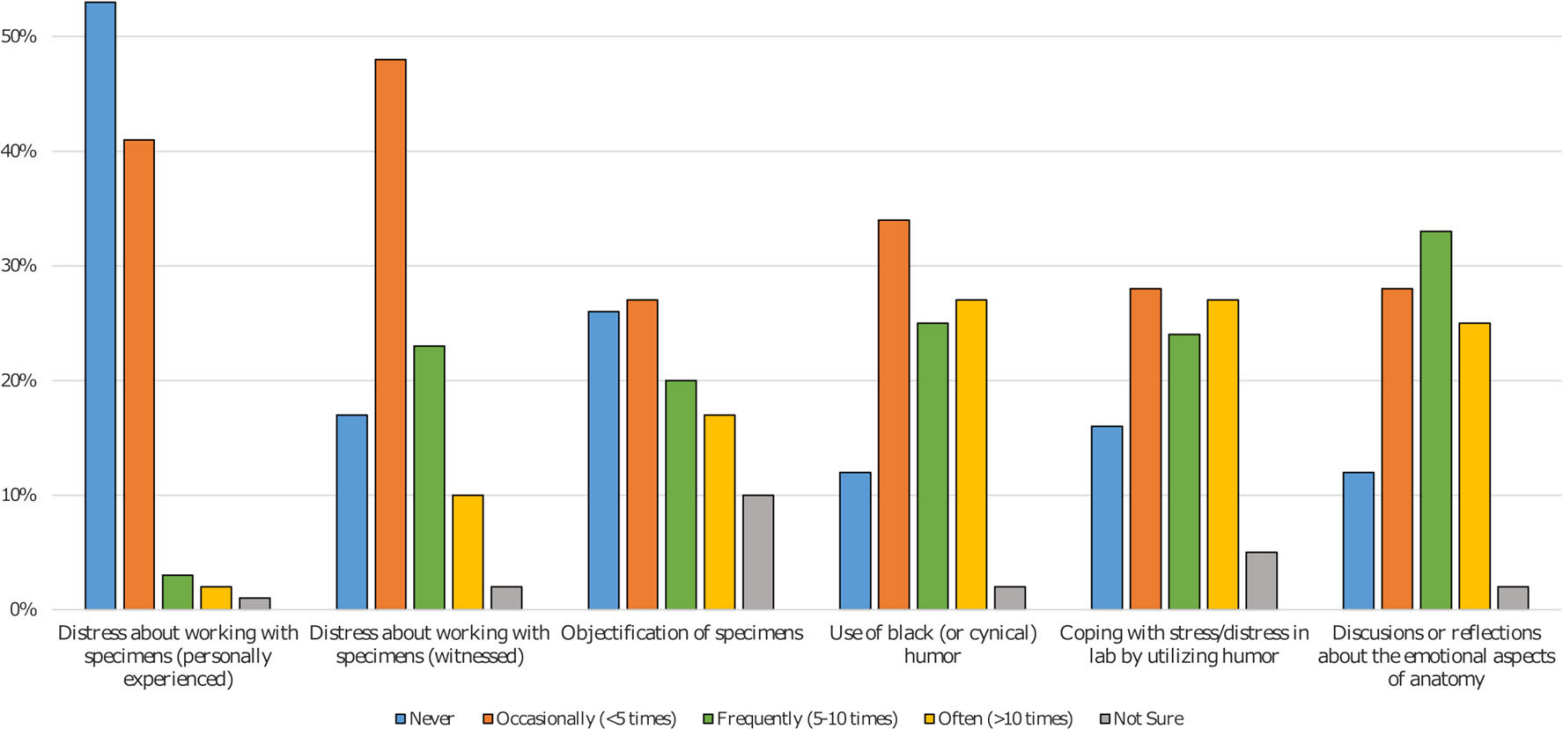
Participant responses to frequency statements indicated that witnessed or personal distress about working with cadaveric specimens was not a common occurrence. However, use of black or cynical humor in labs was experienced at higher rates, as was general use of humor to cope with stress and distress. While objectification of specimens appeared to be not an uncommon experience, many participants also noted that discussions and reflections of the emotional aspects of anatomy were reported to happen frequently

A portion (34%) of respondents reported experience of such dark humor only on occasion, and 27% reported humor to be an “often” experience. Interestingly, 27% of participants also reported that they often experienced moments where humor was used to cope with stress/distress in labs. This was compared with 16% of participants who reported never noticing the use of such a coping mechanism in labs and 12% who reported never noticing black humor at all. Figure 1 depicts all responses for the frequency statements. Also, of note, the majority of participants (86%) reported that discussions or reflections about the emotional aspects were an “occasional” to “often” occurrence for them.

When coding the closed-open combination question, asking if participants had ever heard or used black humor employed in anatomy labs, 72% of participants reported they “Yes,” 20% reported “No,” and 8% were “Unsure” or did not disclose. Of the 72% who acknowledged dark humor in labs, only 16% reported using it, 15% both using and hearing it, and 41% only hearing it.

In response to the presence of humor, data was also analyzed to examine whether any correlations between country or age were reflected in the data. Compared with overall participant percentages, for the top 3 reported countries in our study, each had only about a 50% rate for acknowledging hearing and/or using humor in anatomy labs with 54% for the UK, 56% for the USA, and 53% for Canada. However, it should be noted that the number of respondents from the USA (*n* = 32) and Canada (*n* = 15) were less than those from the UK (*n* = 120), so further meaningful comparisons were not possible. In regard to age, all age ranges also reported similar rates of acknowledging “Yes” to hearing and/or using humor in anatomy labs, with 78% for 18–22 years old, 73% for 23–27, 71% for 28–32, 69% for 38–42, 75% for 43–47, and 64% for the 48+ group.

Specific themes related to humor experiences were also identified from open-ended elaborative responses.

### Content of Humor: from Appetite to Actions

A large number of participants reported the most common use of what they perceived to be “black humor” was the frequent comparison or mention of food in labs. Many also reported that common jokes also involved the acts of dissection, specifically, such as “situations where we have had to hemisect the bodies we have used humor… on using the saws.” Some elaborations were quite dark, as one participant commented, “sometimes there’s jokes of self-harm or suicide with the equipment.” More extensive quotes are highlighted in Table 2.

**Table 2:** The what and why of humor

Theme	Illustrative quotes
Related to content of humor	
Food	“In my experience most black humor comes in the form of comparison of the tissue to other things; normally food. For example, students would compare a muscle to “pulled pork” or fat to cheese.”
	“Lymph looking like black beans and laughing about how we will not be able to eat it again.”
Acts of dissection	“The technician was holding a skin flap, using a clamp, to allow me to dissect. The clamp slipped and the skin fat hit them in the face. We both laughed at this quite a bit. I this dissolved the nervous tension…”
Why use humor	
To cope with morbidity	“I sometimes hear and use black humor when in the anatomy lab… I think this is a coping mechanism to seeing such explicit and graphic imagery live. I have not thought much about it but I do tend to feel less heavy when there is humor around the lab.”
	“It seems to help build a bit of that emotional disconnect that’s necessary to perform something that’s morbid and gruesome like human dissection.”
	“Sometimes the job of [an] anatomist involves gruesome or “unnatural” interactions with deceased humans, and making light of the situation through humor almost acts like a coping mechanism.”
	“Dark/offensive humor can be used as a tool to overcome difficult situations, particularly ones regarding mortality. If it helps those undertaking the dissection get through the task, why not?”
To cope with stress	“Given the intensity of the situation [dissection] for the students involved that this may be a form of coping mechanism and reciprocal chuckling is a way to release tension.”
	“Wanting to lighten the mood, even in anatomy, is perfectly acceptable.”
	“Under immense stress, and doing an activity that is not “normal,” humor seems to be the only go to way to lighten the mood and encourage the team to get on with the work [dissecting].”

### Why Use Humor? Morbidity as Justification

It seems that to many participants, the nature of anatomical lab work made dark humor a natural facet of the work, as highlighted in Table 2. As one participant noted, “we joke to make some of the dissection less morbid,” and another commented that if one was not conscious of anatomy lab actions, it was easy to get “caught in a rabbit hole of morbidity.” However, in addition to dealing with morbidity, some participants noted that occupational humor associated with the lab did not always have to be dark in nature. Puns and anatomical word play were often brought up as an example of extremely common humor, though not considered to be “dark.”

### Inappropriate Humor

As reported, 20% of respondents declined the presence of dark humor in labs when asked if they had ever heard or used it. As one noted, “I seek to maintain a professional face in teaching and the students themselves are always very professional.”

But beyond those who did not acknowledge humor, there was a large proportion of respondents who admitted that dark humor could be viewed as acceptable, given that it did not “cross a line.” Particularly, many participants reported being quite sensitive to humorous comments that could be perceived to be at the “expense” of a donor; donor respect was frequently regarded as a key tenet of anatomy labs.

### Gauging Appropriateness: the Use of “Barometers”

This theme pertained to the metric participants described when assessing the appropriateness of humor within the anatomy laboratory, further detailed in Table 3. This barometer had two manifestations: the internal barometer and the external barometer. The barometer was described almost like a process or flowchart, with objective “yes/no” decisions made along the way.

**Table 3:** Views on “Appropriateness” of humor

Theme	Illustrative quotes
Inappropriate humor	
Disrespect towards donor	“...the line is about respect for the cadaver, it’s normal to make jokes and use black humor in the lab, but I would never joke about a cadaver…”
	“I think it is extremely important to respect the cadavers, and realize the great gift that was given to help us learn. However, since there is a massive responsibility on the students to get a certain grade, whatever aids that help them memorize things or helps them get through the class are fine for them to use as long as they maintain the respect for the body”
(Black) humor is never appropriate	“I think it is NOT appropriate at all times to use humor or show lack of respect towards the donor”
	“I do not believe it [humor] to be appropriate in an anatomy lab setting, being respectful to those who have donated their bodies to science is vital for anatomists”
Gauging the appropriateness of humor	
Internal barometers	“as long as the humor is not offending or making the living uncomfortable (and not about the donor on the table) and learning is happening... I guess this is acceptable?”
	“I think the anatomy lab is a remarkable place to practice self-editing and self-awareness and evaluate the impact our words and actions may have on others. The act of dissection already objectifies the donors to a degree that is beyond my personal comfort level, but it is necessary and it is done carefully, cautiously, and respectfully for a purpose”
	“I would never joke about a cadaver or say anything about it that I would not be comfortable saying about myself or a friend to their face”
External barometers	“I ask myself if I would say/do something in front of a relative or living patient, this helps govern what is acceptable”
	“I think an effective litmus test for occupational/black humor in the anatomy lab is, if this donor was you or your mother, would you appreciate what is being said right now or would it be hurtful?”

The internal barometer was related to participants using self as a gauge for judgments of appropriateness. Instances and internal thoughts included: Would this cause me personal offense? Is this my type of humor? Is the intent malicious?

The external barometer pertained to the impact of external factors when assessing appropriateness. Sub-themes included: Is this occupationally acceptable? Is this appropriate with a living audience? Is this acceptable within my institutional culture? Is there any learning benefit associated with the humor?

### Humorous and “Dirty” Mnemonics

So-called “dirty mnemonics” as a topic indicated another area of polarity among participants. Most acknowledged the use of mnemonics as a whole, although there was high variability in whether participants condoned their use or not, as outlined in Table 4. The influence of internal and external barometric factors appeared to shape the way respondents conditionally condoned this form of dark humor.

**Table 4:** Views on mnemonics

Theme	Illustrative quotes
Use of dirty mnemonics	
Unconditionally condone	“I’ve used a few and they work better because they make you laugh and they suit the dark humor a lot of us have”
	“Dirty mnemonic devices are a hallmark of not only dark humor but undergraduate students in general as well”
Conditionally condone	“I use these devices; they are very effective for learning and I would hate it to be suggested that they are stopped. I have never seen a staff member tell a student a dirty mnemonic but they have been made aware that there are some and that they should go and find their own.”
	“I do not use them personally nor do I tell my students them however I do mention that there are plenty available on the web if they would like to look them up in their own time.”
	“I do use some of them, and I think it is something inherent in medical
	(and health care related professions) that we use black humor to deal
	with the stress. I do think some of them are inappropriate to use in public, but we rely on them to take in a large amount of information as required, and therefore appropriate in a clinical and/or professional setting.”

Responses regarding the use of dirty mnemonics in the anatomy lab yielded opinions ranging from ambivalent to strongly in favor or strongly opposed to their use. The most robust finding from our data on the use of dirty mnemonics is that by far, most respondents conditionally condone their use. Interestingly, the percentage of respondents (16%) who expressly did not condone the use of such mnemonics was roughly the same as those who unconditionally condoned their use (15%). Sixty-nine percent of the responses indicated they would condone the use of these mnemonics based on certain conditions. These conditions represent a subset of data reflecting what circumstances would make such usage appropriate. A significant theme in this subset is that out of those who reported conditional use of dirty mnemonics, many would only do so passively; i.e., would not directly teach the devices, but would hint at where students could discover them on their own.

## Discussion

### Humor Appears in Anatomy Labs as a Means of Coping

Our study confirmed the presence of humor in anatomy labs and highlights some explanations of how and why it is used. This aligns with previously discussed anatomy studies that demonstrated humor to be a means of coping with lab associated distresses [20–22]. Our findings go a step further, by highlighting the use of humor as a coping mechanism for specifically the surreal or morbid acts of dissection. Such findings are supported by broader humor theory. As far back as Freud, humor was theorized to be a mature defense mechanism, to express the feelings of unconscious discomfort [34]. Jokes and laughter are considered by some to provide psychological and physical health benefits [35–37]. And it is theorized that in intense situations, laughter can prove a better means of catharsis than say screaming or breaking down [26]. While anatomy labs are certainly not regarded as emergency scenarios, and the majority of our participants did not report high frequencies of experience or witnessed distress in labs, the same theories might still apply, just on a less extreme scale.

The presence of humor as a means of coping and team bonding is also documented in the clinical components of healthcare and medical education, which aligns better with the educational considerations of anatomy labs [25, 38, 39]. Wear and colleagues provided some more in-depth evidence of how and why dark humor is used, particularly from a medical training standpoint, in a set of focus-group studies with medical students, residents, and attending physicians [23, 24]. Of particular interest to the findings in our study, Wear and colleagues [24] highlight the presence of “unspoken rules” that guide humor in the healthcare setting. These rules include the hierarchy for initiation of humor (i.e., always started by the more senior practitioner), patient populations considered “fair game” (i.e., difficult or non-compliant patients), and limitations to humor. Such limitations included the importance of tone and delivery, as well as respecting “off-limits” patients, such as those with a terminal illness.

Our findings support the presence of similar “rules” in anatomy labs. Whether directly communicated, or an unspoken value, our study indicated that one of the biggest determinants in the importance of humor is regard for the donor. As our illustrative quotes exemplify, anatomical donation being regarded as a true “gift” implies that there is a responsibility of respect towards the donor that might be considered an important “rule” of the anatomy lab. This may be related to the increasingly standardized practice in anatomy education of referring to donors in a more humanistic fashion [4, 5].

### Differences in Opinions

Our results also highlight that there is a subset of individuals who believe that maintaining “professional behavior” in labs means that humor is never appropriate to use. In our open-closed coding question, 20% of respondents put they had never heard or used dark humor in the lab. Our frequency statement analysis revealed that 12% of participants put they had “Never” experienced the use of black (cynical) humor in the lab.

The differences in these responses may be explained by the wording of the question, with a greater percentage denying humor when asked to elaborate on their views. However, if the range can be considered 12–20% of individuals who believe that this type of humor simply does not exist, it begs the question as to why they are at odds with the majority. Upon examining these response rates compared with demographic information provided, there appeared to be no significant trends in any particular group and the comments that humor is considered “inappropriate.” Therefore, our study does not indicate what the implications from such stark differences in professional opinions may be. However, we hypothesize that such strong professional stances may also shade teaching and learning opinions.

For example, when asked about views on “dirty mnemonics,” a humorous approach to learning/recalling information, participants were split in responses. A review of the literature suggests that this concern is related to the pervasive use of acronyms in healthcare as a whole [40, 41], since a widespread reliance on potentially confusing acronyms has been identified as inherently problematic [42]. Such rapid and voluminous recall has an especially rich tradition in the classical study of anatomy [43]. But this very tradition points to the even greater need for future physicians to build a deeper fundament of long-term understanding of medical and anatomical concepts, rather than mere rote recall of data encoded in letters that could all too easily be accidentally interchanged (with disastrous results). Such depth is key to related issues of professionalism [44].

Less than 2% of respondents denied knowledge of the existence of mnemonics in the anatomy lab. The data showed almost all respondents (nearly 99%) were aware of the use of mnemonics in the anatomy lab. There is a common theme in the belief among respondents that mnemonics are in general, dirty or otherwise, an effective albeit lower-level learning tool that should not feature as a planned teaching device. Polarity appears in how much “ownership” they are prepared to take in the dissemination/tolerance of their use in professional practice, which points back to the internal and external barometer. Many respondents indicated that mnemonics ought to be left to students to discover on their own (as part of the “hidden curriculum”). Other respondents reflected on their own use of mnemonics to memorize structural anatomy, and 12% responded with an unprompted mention of the “cranial nerves mnemonic” as an example. Smith and Border [45] point out that mnemonics “build a construct for subsequent deeper layers of knowledge.” In our study, the difference of opinion on the use of dirty mnemonics in the anatomy lab reflects on issues of identity in professional practice.

### Humor as a Hidden Curriculum of Anatomy Labs

Given our findings in the variability of humor experiences and questions that arise from such diversity, our study confirms that humor is a facet of the hidden curriculum of anatomy labs. It also highlights the need for more recognition of the hidden curriculum in anatomy. As Hafferty and Finn [46] highlight, the anatomy lab, in particular, can be considered a space for professional formation, related to hidden curriculum. As they describe it, the hidden curriculum can be considered the differences in what an organization says and what it actually does, as well as the non-formal aspects of organizational function. In considering the anatomy lab as an “organization,” the hidden curriculum is demonstrated by the presence of humor in this study. Many participants noted that while humor was not often directly and openly recognized, it still persisted, as a facet of organizational structure. The power of humor within the anatomy lab, whether it be positive or negative, stems from its often-tacit nature.

The hidden curriculum is often linked to implications for professional practice. As Escobar-Poni and Poni [47] highlight, there is an opportunity for the gross anatomy curriculum to play a major role in professionalism-related training for medical students. Their article highlights particular curricular learning activities which may facilitate professional development in gross anatomy, while also emphasizing the need for peer review evaluations of such activities. Swick [48] also highlights some professionalism aspects of anatomy experiences, such as adherence to ethical and moral standards, demonstrating humanistic values, and dealing with complexity/uncertainty that might be considered more hidden rather than formal curriculum. Both of these articles make excellent arguments for more professionalism-focused research and evaluation in the anatomical sciences. And as our study highlights, there may be interesting findings for those who instruct the anatomical and basic sciences, such as the argument for more direct discussions of the hidden curriculum.

### Limitations, Considerations, and Future Directions

While our study highlights that humor is widely used by many individuals in anatomy labs, there are several considerations that should be recognized as limitations of our findings.

First, while our study attempted to recruit a wide scope of international participants, it should be noted that it was written in British English. Only approximately 3% of participants reported their country of residence to be a country where English is not the predominant language used in higher education institutes. Language and cultural differences can be considered a limitation of this study from a truly international perspective. Even the concept of humor is a complex cognitive process that can be influenced by an individuals’ culture [49]. This thus limits a true cross-cultural understanding of humor utilization in anatomy labs, particularly when considering the native language and cultures (American and British) of the research team. Future directions for similar studies might include international collaboration or use of formal translation services for recruitment and data collection. Language variances could also have inadvertently resulted in a conflation of humor with jokes and laughter, as humor can be a nuanced concept. This could be mediated and better understood in future studies by asking individuals to also self-report their definition of “humor,” and subsequently black/dark humor, in order to better identify potential discrepancies in communication.

The limitations of recruitment methods should also be considered in our comparison of international and age groups. The sampling methods used let to a wide demographic range, which made in-group comparisons difficult. Our findings suggest that the presence of humor was reported to be roughly the same across our top three countries of responses, as well as across age groups. However, given recruitment tactics for our study, the resulting participant totals displayed homogeneity in age and country of residence. An additional future direction would be to use more purposeful sampling methods, instead of snowball recruitment, to see if the similarity in responses is still apparent when the overall sample is more heterogeneous in its demographics.

Another couple of considerations we wish to note were related to the occasional ambiguity of our study design. First, we did not require participants to specifically define their individual definitions of terms such as “stress,” “distress,” or even “black humor.” This flaw in survey design may explain subtle differences in reports of the use of humor or even stress experienced in the lab, as we see between our frequency statements and open-ended responses. In the absence of shared understanding, it could be argued that the minutia of stress and humor views cannot be concluded from the present study. However, we do believe that the general views and trends are well enough reported to be considered supportive evidence on broad views of humor and stress in anatomy labs.

Second, as highlighted in our survey details and results, frequency statements were based on numeric values determined by the research team to provide some context, but not limit participation. This presents the limitation of the possible confounding factors related to time that may be reflected in our data. For example, to a participant who has been teaching anatomy for 15+ years (reported by numerous respondents), hearing something “Often,” or more than 10 times, may be interpreted quite differently than an “Often” rating reported by a year 1 health professions student with limited lab experiences. While it is exponentially challenging to attempt to account for such considerations without limiting inclusion criteria, we do not think these discounts our results.

Our results provide strong evidence to confirm the presence of hidden curriculum in anatomy labs [46] and encourages more specific research into subdivisions of this curriculum, often related to the emotional, professional, and ethical considerations of anatomy. We suggest and encourage more specified work be targeted at these potential curricular components, to best understand how curricula are being implemented, and the benefits. It may also be key to investigate the curricular decisions to not include more humanistic aspects. For example, our results highlight that about 12% of respondents stated that they never have experienced discussion or reflections about the emotional aspects of anatomy. Could this be due to the documented restraints of contact hours in many anatomy courses [50–53] or is this a personal decision made by these individuals? Perhaps, while there has been an increase in donor-focused activities [54–56], not all institutions host or focus on such humanistic activities or do not require them to be mandatory for students and staff. Further research might allow us to better understand the breadth and differences we as anatomy educators certainly possess.

## Conclusion

Humor is widely regarded to be a coping mechanism in anatomy labs, particularly when dealing with what are regarded as “morbid” or “surreal” acts of working with human specimens. However, humor does not reign unconstrained in labs. This study highlights that while dark humor may be a perceived tension release, many individuals make use of very specific internalized gauges to determine when and what humor may be appropriate. And one of the most important of these is that donor respect be of the utmost importance at all times. Still, there are a minority of people who believe that humor is never appropriate in the lab. The dichotomy in professional views indicated in this study highlight the need for future humanistic-focused anatomy education research, to better understand and have ideal educational experiences for all. Finally, this study further highlights the impact of the hidden curriculum associated with the use of humor with educational and professional settings.
